# 

**Published:** 1899-07

**Authors:** 


### INDEX TO VOL. XV.

Abortion; Spontaneous and Induced....................... 472
Abstracts and Selections......37, 148, 209, 261-278, 317-332, 387-394, 435-452.................................537-540
A Campaign of Education................................ 637
A Clash of Authority in Quarantine Matters............... 541
A Communication to the Chief Surgeon of the Department of Santiago and Puerto Principe........................ 507
Alas, Poor Summers!.................................. 105
American Electro-Therapeutic Association ................ 258
A New District Medical Society......................... 583
An Important Circular ................................ 479
An Important Decision................................. 333
A Notable Paper ..................................... 283
An Open Letter to Dr. Hill from Dr. Daniel Parker........ 257
A Pull All Together.................................. 338
Are Dentists Doctors?................................. 378
Arsenical Acids in Chemicals........................... 477
A State Board of Health............................... 543
As to Sewers and Sewage.............................. 281
As to Statistics and Sanitation......................... 279
A Substitute for the Diamond Disk...................... 430
A Texas Doctor who Knows How to Entertain.............. 340
Austin District Medical Society......................... 208
A Walking Case of Typhoid Fever—Perforation and Death... 305

B. E. Hadra, M. D. (Biographical Sketch)................. 633
Bichloride of Mercury Baths in the Treatment of Variola.... 245
Bill Nye and the Nurses................................ 94
Books and Magazines........51, 113, 169, 289-294, 345-347, 409-412, 459, 494-504, 547-548...................643-646
Brazos Valley Medical Association ...................... 316
Brazos Valley Medical Association ...................... 585
Brazos Valley Medical Association — Eighth Semi-Annual Meeting ........................................ 260

Carbolic Acid Poisoning................................ 119
Central Texas Medical Society.......................... 86
Central Texas Medical Association ..................... 629
Circular Letter from the State Health Officer.............. 527
Closure of the Abdominal Incision After Laporotomy and the Tendency of Hernia................................ 93

Delirium Tremens in Moderate Consumers of Alcohol........ 100
Dental Department ............................254-257, 313

Dental Items of Interest.......................... 478
Dental Notes ..............................35, 84, 141
Dental Surgeons in the Armies and Navies................ 132
Dentistry Retrograding ............................ 377
Dentists in the Army and Navy........................ 81
Dr. P. C. Coleman.................................. 544
Dr. Doty, Port Health Officer in New York............... 108

East Texas Medico-Chirurgical Society .................. 630
Electricity and Gynecology............................ 517
Empyema of Pleural Cavity—Report of Four Cases......... 424
Erysipelas ......................................... 366
Eulogy on Dr. R. M. Swearingen........................ 587

H. A. West, M. D. (Biographical Sketch)................ 635
Has the Germ of Yellow Fever Been Found?............... 163
Hearne is Ahead...................................... 13
Hernia—Ferguson's Operation........................... 77
Hit It at Last....................................... 340
Honey-Fuggling the Doctors............................ 39
How to Secure Safe and Successful Vaccination........... 528
How we Apples do Swim............................... 400

Instrumental Delivery................................ 468
International Medical Congress, Paris.................. 315
Intestinal Perforations from Within by Foreign Bodies...... 1
Intestinal Suture with the Use of a Potato Cylinder......... 307
Is Dentistry a New Profession?......................... 140
Is Fever a Conservative Force?......................... 461

Knowledge is Power.................................. 72

Lady Churchill and Mrs. Maybrick...................... 399
Law-Making a la Texas............................... 455
Love's Strategem—The Doctor Puts Up a Job on the Major... 223

Maladministration of Public Medical Affairs in the State of Texas ........................................ 555
"Medical Dogs" ..................................... 46
Medical News and Miscellany.....48, 108, 166, 227, 283-288, 342-344, 404-408, 456-458, 492-494, 545-547, 598-600, 640-643
Minutes of the Regular Meeting of the West Texas Medical Association ....................................... 14
Mr. E. A. Hyde...................................... 107

National Quarantine.................................. 596
New York Academy of Medicine—Meeting of November 17, 1899 ............................................ 372

New York Academy of Medicine—Section in Orthopædic Surgery ........ 18
Notes ........ 482

Only a Diploma Required ........ 336
Ossification of the Choroid ........ 476

Program Mississippi Valley Medical Association ........ 142
Random Dots ........ 184
Rape of the Red Back ........ 164
Rational Treatment of Smallpox ........ 302
Recollections of a Rebel Surgeon and Other Sketches; or, In the Doctor's "Sappy Days" ........ 217
Remarks on Suprapubic Lithotomy ........ 308
Reorganization ........ 584
Revision of the United States Pharmacopœia ........ 44
Richmond Academy of Medicine and Surgery ........ 204

Sanitary Plumbing ........ 183
Sanitary Reform in Texas—Two Prize Papers on Sanitation .. 123
Scalp Wounds ........ 515
Selected Formulæ ........ 161, 288-289
Sequelæ of Typho-Malarial Fever, Complicated with Early State of Opium Habit Established by Medical Attendants. 252
Smallpox in Texas ........ 165
Some Further Observations on the Echinococcus Disease, or Bladder Tapeworm ........ 415
Some Observations on the Echinococcus Disease, Especially as Related to the Present Epidemic Among the Prairie Rabbit ........ 237
Some Recent Cases of Appendicitis ........ 175
Some Statistics and a Moral ........ 432
Some Tonsil Clippings ........ 5
Something Needed for the Worthy Doctor to be Appreciated ... 466
Stone in the Bladder and Lithotomy ........ 603
South Texas Medical Association ........ 586
South Texas Medical Association ........ 26
Superintendent's Worsham's Report ........ 401
Sympathetic Ophthalmia ........ 138
Synopsis of Crown and Bridge Work ........ 429

Texas Dental Association ........ 537
Texas Epileptic Asylum—Dr. Worsham's Report ........ 97
Texas State Medical Association ........ 482
Texas State Medical Association—Call of President ........ 529
Texas State Medical Association—Program of Annual Meeting ........ 530
That $10,000 Library ........ 491
The Baby's First Week ........ 364

The Central Texas Medical Association .................. 434
The Collapse of a College.............................. 594
The Death Rate from Nephritis in 140 Cities in the United States, for 1898.............................. 355
The Good Die Young..................................... 339
The Evils of Present Methods of Education................ 297
The First Stage of Labor................................ 368
The Hair of the Dog..................................... 164
The Ideal Doctor ....................................... 420
The Importance of Nasal Breathing in Early Childhood..... 250
The Lawyer and the Doctor............................... 61
The President-elect .................................... 597
The Proposed Health Bill................................ 523
The Public Health....................................... 146
The Quarantine Laws of Texas and Other Items............ 187
The Quack's Paradise.................................... 485
The Report of the Health Officer in San Antonio........... 107
The Squamous Palmar Syphilide........................... 135
The Teeth of Races...................................... 379
The Texas Insane Asylums................................ 402
The Texas Quarantine Law and Other Matters.............. 218
The "Texas State Board of Health"...................... 165
The Texas State Medical Association .................... 434
The Wise Men of Texas................................... 41
Thirteenth Semi-Annual Meeting of the Central Texas Medical Association ......................................... 381
Three Cases of Closure of Jaws, Produced by Mercurial Stomatitis ............................................ 563
Thirty-second Annual Meeting of the Texas State Medical Association ..............................566-616, 616-629
Treatment of Yellow Fever............................... 509
Traumatic Surgical Condition of the Bladder and Treatment.. 612
Twenty-second Annual Meeting Texas State Medical Association ................................................ 544
Typhoid Fever as a Systemic Disease of Manifold Manifestations ............................................. 95

Urinalysis ............................................. 359

Vaccination ............................................ 512
Vivisection: The Anti-Vivisection Society............... 395

Western Texas Medical Association ...................... 194
Western Texas Medical Association ...................... 259
Western Texas Medical Association ...................... 385
Western Surgical and Gynecological Association........... 258
"What Would Jesus Do?"................................. 453

Yellow Fever in 1899 in the South....................... 341

